# Implementation of sepsis' treatment protocols in an emerging country from 2005-2014: an analysis of 21,103 patients

**DOI:** 10.1186/2197-425X-3-S1-A219

**Published:** 2015-10-01

**Authors:** FR Machado, LC Pontes de Azevedo, EMF Ferreira, J Lubarino, C Silva, P Schippers, A Pereira, IC de Paula, BF Mazza, MC Assumpcao, H Fernandes, N Akamine, R Salomao, E Silva

**Affiliations:** Latin American Sepsis Institute, Sao Paulo, Brazil

## Intr

Previous studies already showed a reduction in sepsis' mortality rates after the implementation of protocols based on the Surviving Sepsis Campaign (SSC) bundles, in high income countries. However, there is no similar study in emerging settings.

## Objectives

To assess the impact of a national initiative in implementing sepsis protocols in Brazilian institutions, analyzing them according to the source of income (public or private).

## Methods

Retrospective analysis of the Latin America Sepsis Institute (LASI) database, from 2005 to 2014. Participation was voluntary. The implementation process was based on a multifaceted intervention including a local sepsis team, protocols, screening procedures, laboratory and antibiotics flowchart for emergency department (ED), wards and intensive care units (ICU), checklists, physicians and nurses training nd audit/feedback strategies. After the initial training, the institutions collect data on SSC bundles compliance and hospital outcome in patients with severe sepsis or septic shock in all hospital settings. We included only the institutions with at least 80 patients and at least one year of data collection, excluding patients admitted after the first four years of the campaign. All patients were followed until hospital discharge. We define public institutions as those with the major income coming from public sources and private as those coming from private insurances.

## Results

We included 21,103 patients from 65 institutions being 9,032 from public institutions and 12,071 from private ones. Comparing the 1^st^ semester with the 8^th^ semester, compliance with the 6-hours bundle increased from 13.5% to 58.2% in the private institutions while the public ones improved from 7.4% to 15.7%. Mortality rates significantly decreased throughout the program in private institutions (1st semester: 47.6%, 8^th^ semester: 27.2%; odds ratio (OR): 0.45; 95% confidence interval (CI): 0.32-0.64). However, there is no significant reduction in the public institutions throughout the semesters (1^st^ semester: 61.3%; 8^th^ semester: 54.5%, OR: 0.63; 95%CI: 0.39-1.02). The intervention reduced the mortality rates throughout the semesters in patients from all settings (1^st^ semester vs 8^th^ semester: ED - OR: 0.55; 95%CI: 0.38 - 0.79; wards - OR: 0.59; 95%CI: 0.42-0.83; ICU - OR: 0.46; 95%CI: 0.39 - 0.54) although the effect was less consistent in the ICU. In patients from private ED, mortality rates decreased from 38.1 to 21.2% (p < 0.001) while in the public institutions this reduction was not significant (56.3% to 49.8%, p = 0.057).

## Conclusions

The implementation of sepsis protocols resulted in improved compliance to the quality indicators and reduction in mortality rates. The impact of this intervention was different in public and private institutions.
